# Characteristics and outcomes of hospitalized patients with cardiovascular complications of COVID-19

**DOI:** 10.34172/jcvtr.2021.53

**Published:** 2021-12-06

**Authors:** Farzad Jalali, Farbod Hatami, Mehrdad Saravi, Iraj Jafaripour, Mohammad Taghi Hedayati, Kamyar Amin, Roghayeh Pourkia, Saeid Abroutan, Mostafa Javanian, Soheil Ebrahimpour, Niloufar Valizadeh, Saeede Khosravi Bizhaem, Naghmeh Ziaie

**Affiliations:** ^1^Department of Cardiology, Babol University of Medical Sciences, Babol, Iran; ^2^Cardiovascular Diseases Research Center, Birjand University of Medical Sciences, Birjand, Iran; ^3^Infectious Diseases and Tropical Medicine Research Center, Health Research Institute, Babol University of Medical Sciences, Babol, Iran; ^4^Clinical Research Development Unit, Razi Hospital, Birjand University of Medical Sciences, Birjand, Iran

**Keywords:** COVID-19, Cardiovascular Diseases, Mortality, Risk, Complications

## Abstract

***Introduction:*** To address cardiovascular (CV) complications and their relationship to clinical outcomes in hospitalized patients with COVID-19.

***Methods:*** A total of 196 hospitalized patients with COVID-19 were enrolled in this retrospective single-center cohort study from September 10, 2020, to December 10, 2020, with a median age of 65 years (IQR, 52-77). Follow-up continued for 3 months after hospital discharge.

***Results:*** CV complication was observed in 54 (27.6%) patients, with arrhythmia being the most prevalent (14.8%) followed by myocarditis, acute coronary syndromes, ST-elevation myocardial infarction, cerebrovascular accident, and deep vein thrombosis in 15 (7.7%), 12 (6.1%), 10(5.1%), 8 (4.1%), and 4 (2%) patients, respectively. The proportion of patients with elevated high-sensitivity troponin I, N-terminal pro-B-type natriuretic peptide, left ventricular diastolic dysfunction, and heart failure with preserved ejection fraction was greater in the CV complication group. Severe forms of COVID-19 comprised nearly two-thirds (64.3%) of our study population and constituted a significantly higher share of the CV complication group members (75.9%vs 59.9%; *P* =0.036). Intensive care unit admission (64.8% vs 44.4%; *P* =0.011) and stay (5.5days vs 0 day; *P* =0.032) were notably higher in patients with CV complications. Among 196patients, 50 died during hospitalization and 10 died after discharge, yielding all-cause mortality of 30.8%. However, there were no between-group differences concerning mortality. Age, heart failure, cancer/autoimmune disease, disease severity, interferon beta-1a, and arrhythmia were the independent predictors of all-cause mortality during and after hospitalization.

***Conclusion:*** CV complications occurred widely among COVID-19 patients. Moreover,arrhythmia, as the most common complication, was associated with increased mortality.

## Introduction

Although it has been a year since the new coronavirus, named SARS-CoV-2, emerged and caused the devastating outbreak of COVID-19, the infection and mortality rates are on the rise. COVID-19 is known by its pulmonary manifestation, and acute respiratory distress syndrome (ARDS) is considered as the main cause of mortality. However, soon after, a growing body of literature directed attention to cardiac involvement. Underlying cardiac disease or hypertension is shown to be the major risk factor of poor prognosis.^1-3^ Recent reports have indicated that a considerable portion of patients experience a cardiac injury, ranging from 7.2% to 22.2%, which is linked to higher mortality.^3^ Nevertheless, previous studies have exclusively focused on the cardiac injury defined as a raised cardiac marker without a definitive diagnosis. To our knowledge, the present retrospective cohort study is the first study to comprehensively address cardiovascular (CV) complications and related outcomes in COVID-19 patients.

## Materials and Methods

### Study design and participants

The current single-center retrospective cohort study was conducted at Rohani Hospital of Babol, Iran, which is a designated hospital by the Babol University of Medical Sciences to treat cases with COVID-19. The local university’s ethics committee approved the study protocol, and informed consent was obtained from participants on admission. The study recruiting period was from September to December 2020, and 196 patients participated in this study. We enrolled adult patients admitted to our hospital with a confirmed diagnosis of COVID-19 and a consultation requested from the cardiology department. A confirmed diagnosis of COVID-19 is defined as a positive result of the Reverse Transcriptase-Polymerase Chain Reaction (RT-PCR) test for a nasal or nasopharyngeal swab specimen. Reasons for requesting a consultation were raise of cardiac markers, dyspnea, electrocardiography (ECG) changes, chest pain, and underlying cardiovascular diseases in 53 (27%), 34 (17.4%), 19 (9.7%), 13 (6.6%), and 4 (2%) cases, respectively. Also, the reason of consultation was not mentioned in 73 (37.3%) cases. All decisions for requiring consultations in the current study were made by a single attending infectious disease specialist and were provided by a single cardiologist. The decision for whether or not to request a consultation from a cardiologist was based on developing signs and symptoms related to the CV system, alterations in cardiac markers, ECG findings, and, eventually, the clinical judgment of the attending physician. Patients who left the hospital against medical advice or had incomplete data were excluded.

### Data collection

The electronic medical records were used by two independent expert investigators to review and collect demographics and clinical and laboratory findings on admission, in-hospital treatments, and outcomes. Cardiac examinations included cardiac biomarkers, electrocardiography, and echocardiography. Data regarding complications during hospitalization were extracted, and patients were categorized into two groups concerning the presence or absence of CV complications. Indications for echocardiography have been defined by the Iranian Society of Echocardiography as following: shock, new arrhythmia, cardiomegaly or pericardial effusion observed in computed tomography, elevated cardiac markers, significant changes in ECG, generalized edema, and new clinical alterations unexplainable by COVID-19. All transthoracic echocardiographic (TTE) assessments were performed by a single cardiologist, who was provided with personal protective gear according to national guidelines. The dedicated ultrasound machine for COVID-19 patients was a General Electric Vivid 7. A Cardiac Magnetic Resonance imaging was performed for patients with high suspicion of myocarditis. Cardiac biomarkers and ECG findings were collected on the same day of TTE.

### Definitions and Outcomes

We evaluated both CV and non-CV complications during the time of hospitalization. We have only considered patients with new-onset cardiovascular structural and/or functional abnormality during their hospital course in the CV complication group. New echocardiographic changes during hospitalization had been distinguished from preexisting abnormalities by the cardiologist. In all cases, we distinguished preexisting and existing abnormalities by reviewing the patient’s medical records including the latest ECG or echocardiography related to the time before hospitalization. CV complication category included ST-Elevation Myocardial Infarction (STEMI) defined as an elevation in cardiac biomarkers and ST-segment with the further establishment of angiography results, other acute coronary syndromes (ACS), new-onset arrhythmia, deep vein thrombosis (DVT), cerebrovascular accidents (CVA), and myocarditis. Heart failure with preserved ejection fraction (HFpEF) was defined by signs and/or symptoms of HF with a left ventricular ejection fraction (LVEF) ≥50% and normal ventricular size along with evidence of abnormal LV filling and elevated filling pressures based on imaging or laboratory findings.^4^ Criteria for diagnosis of a clinical case of myocarditis were signs and/or symptoms with changes in cardiac biomarkers or echocardiographic findings suggestive for myocardial inflammation in case of absence of coronary involvement using invasive or non-invasive imaging methods. Reference is cited at the end in the manuscript.^5^ Non-CV complications included ARDS, acute kidney injury (AKI) as per Berlin and Improving Global Outcomes definitions, respectively, and sepsis. Patients with a respiratory rate of ≥ 30 breaths/min, resting fingertip oxyhemoglobin saturation ≤93%, shock, and those who required ICU admission or inotropic administration were defined as severe cases according to the national guideline for the management of COVID-19 patients (version 9) released by the National Ministry of Health of Iran. Our primary outcome of interest was the overall all-cause mortality of COVID-19 patients. Secondary outcomes of interest were ICU admission, hospital stay, need for invasive and/or non-invasive ventilation, and readmission. Patients who showed improved clinical signs and symptoms with normal body temperature and resolved radiographic features with two consecutive negative results of RT-PCR for COVID-19 fulfilled the discharge criteria and left the hospital. The follow-up period for out-of-hospital mortality, complications, and readmissions was three months after hospital discharge.

### Statistical analysis

Continuous variables are presented as mean ± standard deviation (SD), while the categorical data are presented as numbers and percentages. We used the χ2 and fisher’s exact tests for comparison between categorical variables in each group. A Cox regression analysis was utilized to determine the independent impact of each of the variables for predicting primary outcome of interest. Survival curves were analyzed through the Kaplan-Meier method. A two-sided P-value of less than 0.05 was considered to be statistically significant. Statistical analyses were performed using SPSS version 22.0 software (SPSS, Inc., Chicago, IL, USA).

## Results

### Baseline patient characteristics

A total of 196 hospitalized adult patients with confirmed COVID-19 were enrolled including 54 cases in the CV complication group and 142 cases in the non-CV complication group.

The median age was 65 (IQR, 52-77) years, and men constituted 53.1% of the study population. Demographic values regarding age and sex were not significantly different between the two groups (Table 1).

**Table 1:** Baseline demographic, clinical, and laboratory characteristics of 196 patients with COVID-19

**Characteristics**	**Total (n=196)**	**Non-CV complication (n=142)**	**CV complication (n=54)**	***P*** **-value**
Age, median (IQR), y	65 (52-77)	65 (54.25-76.75)	65 (43.25-77.5)	0.713
Male, No. (%)	104 (53.1)	73 (51.4)	31 (57.4)	0.452
**Comorbidities**				
Diabetes, No. (%)	34 (17.3)	22 (15.5)	12 (22.2)	0.266
Hypertension, No. (%)	105 (53.6)	79 (55.6)	26 (48.1)	0.348
Coronary artery disease, No. (%)	37 (18.9)	29 (20.4)	8 (14.8)	0.370
Heart failure, No. (%)	47 (24)	35 (24.6)	12 (22.2)	0.722
Chronic obstructive pulmonary disease, No. (%)	17 (8.7)	13 (9.2)	4 (7.4)	1
Cancer/autoimmune disease, No. (%)	13 (6.6)	12 (8.5)	1 (1.9)	0.118
Chronic kidney disease, No. (%)	7 (36)	5 (3.5)	2 (3.7)	1
Number of comorbidities				
0	40 (20.4)	29 (20.4)	11 (20.4)	0.111
1	67 (34.2)	42 (29.6)	25 (46.3)	
2	74 (37.8)	60 (42.3)	14 (25.9)	
3	15 (7.7)	11 (7.7)	4 (7.4)	
**Clinical Presentation**				
Dyspnea, No. (%)	129 (65.8)	98 (69)	31 (57.4)	0.126
Chest pain, No. (%)	5 (2.6)	2 (1.4)	3 (5.6)	0.129
Fever, No. (%)	38 (23.8)	31 (25.4)	7 (18.4)	0.377
Systolic blood pressure, mean (SD), mmHg	117.5 (106.25-138)	115 (110-130)	120 (100-140)	0.519
Heart rate, median (IQR)	82 (76.25-100)	82 (78-99)	84 (72-100)	0.715
Respiratory rate, median (IQR)	20 (18-23.5)	20 (18-23)	20 (18-24)	0.982
Blood oxygen saturation, median (IQR), percent	93 (88-96)	93 (88-96.5)	93 (88-96)	0.771
**Medication History**				
Aspirin, No. (%)	76 (38.8)	56 (39.4)	20 (37)	0.758
Atorvastatin, No. (%)	75 (38.3)	55 (38.7)	20 (37)	0.827
angiotensin-converting enzyme inhibitor/angiotensin receptor blocker, No. (%)	74 (37.8)	56 (39.4)	18 (33.3)	0.431
Anticoagulant, No. (%)	23 (11.7)	15 (10.6)	8 (14.7)	0.409
Furosemide, No. (%)	32 (16.3)	24 (16.9)	8 (14.8)	0.724
Novel oral anticoagulant, No. (%)	4 (2)	3 (2.1)	1 (1.9)	1
**Laboratory**				
Leukocytes, median (IQR), /μL	9800 (7450-13050)	9700 (6800-12525)	10100 (8000-13900)	0.150
Platelets, median (IQR), ×10^3^/μL	208 (154-282)	212(154.5-277.2)	192 (153-310)	0.798
Erythrocyte sedimentation rate, median (IQR), percent	45 (25-82.75)	50 (22.5-82.5)	70 (38-100)	0.036^*^
Creatinine, median (IQR), mg/dL	1.2 (0.85-2)	1 (0.85-1.7)	1.3 (1-2)	0.181
Blood urea, median (IQR), mg/dL	27 (16-49.25)	25 (16-47)	34 (25-58)	0.065
Raised high-sensitivity Troponin I, No. (%)	27 (13.8)	5 (3.5)	22 (40.7)	<0.001
C-reactive protein, median (IQR), mg/dL	70 (19-123.5)	71 (20.5-145)	140 (67-190)	0.041^*^
Raised N-terminal pro-B-type natriuretic peptide, No. (%)	141 (72)	96 (67.6)	45 (83.3)	0.029^*^
Procalcitonin, median (IQR), ng/mL	0.5 (0.1-2.7)	0.5 (0.1-3)	0.5 (0.18-3.5)	0.295
Interleukin-6, median (IQR), pg/mL	45 (14-126)	41 (14-120.5)	46 (13.5-148.25)	0.675

Abbreviation: IQR, interquartile range.

The most prevalent symptom was dyspnea (65.8%), and the incidence rate of fever was 23.8%. The median oxyhemoglobin saturation was 93% (88-96) among the patients. These and other clinical signs and symptoms were comparable in the two groups.

Of the comorbidities, hypertension was the most frequent comorbidity accounting for more than half of the participants in our study (53.6%). There was no significant difference between the two groups regarding the type or number of comorbidities. We also analyzed outpatient medications used for more than three months before admission and found no significant difference between the groups.

### Laboratory findings and treatments

The median amounts of inflammatory biomarkers, including erythrocyte sedimentation rate (ESR) and C-reactive protein (CRP), were significantly higher in patients with CV complication than those without CV complication (*P* = 0.036 and *P* = 0.041, respectively). Cardiac injury as defined by high-sensitivity troponin I (hs-TnI) > 99th percentile upper reference limit was present in 13.8% of all patients and was notably more frequent in patients with CV complication compared to patients without CV complication (40.7% vs 3.5% *P* < 0.001). A similar result was obtained when comparing cases with an elevated level of N-terminal pro-B-type natriuretic peptide (NT-proBNP) ( > 125 pg.mL-^1^) between the two groups (*P* < 0.029) (Table 2). The descriptive results of ferritin, D-dimer, and lactate dehydrogenase were only reported in 50, 81, and 98 patients, respectively (Table 3).

**Table 2:** In-hospital treatment, electrocardiographic, and echocardiographic data and clinical outcomes of 196 patients with COVID-19

**In-hospital treatment**	**Total (n=196)**	**Non-CV complication (n=142)**	**CV complication (n=54)**	***P*** **-value**
Inotropic, No. (%)	39 (19.9)	23 (16.2)	16 (29.6)	0.035^*^
Lopinavir / Ritonavir, No. (%)	70 (35.7)	52 (36.6)	18 (33.3)	0.668
Hydroxychloroquine, No. (%)	157 (80.1)	109 (76.8)	48 (88.9)	0.057
Azithromycin, No. (%)	28 (14.3)	21 (14.8)	7 (13)	0.744
Remdesivir, No. (%)	1 (0.5)	0	1 (1.9)	0.276
Clopidogrel, No. (%)	31 (15.8)	20 (14.1)	11 (20.4)	0.281
Heparin, No. (%)	133(67.9)	96 (67.6)	37 (68.5)	0.903
Furosemide, No. (%)	102 (52)	68 (47.9)	34 (63)	0.059
Interferon, No. (%)	79 (40.3)	56 (39.4)	23 (42.6)	0.687
angiotensin-converting enzyme inhibitor/angiotensin receptor blocker, No. (%)	26 (13.3)	20 (14.1)	6 (11.1)	0.583
Aspirin, No. (%)	53 (27)	29 (20.4)	24 (44.4)	0.001^*^
Novel oral anticoagulant, No. (%)	1 (0.5)	0	1 (1.9)	0.276
Antibiotics, No. (%)	42 (21.4)	31 (21.8)	11 (20.4)	0.824
Atazanavir, No. (%)	58 (29.6)	38 (26.8)	20 (37)	0.159
**Electrocardiographic findings**				
QRS complex abnormality, No. (%)	28 (14.3)	21 (14.8)	7 (13)	0.744
ST-segment changes, No. (%)	39 (19.9)	21 (14.8)	18 (33.3)	0.004^*^
QT interval abnormality, No. (%)	9 (4.6)	6 (4.2)	3 (5.6)	0.709
QT interval duration, median (IQR), millisecond	402 (360-443)	402 (360-434)	405 (354-468.75)	0.676
**Echocardiographic findings**				
LV Dilation, No. (%)	22 (11.2)	16 (11.3)	6 (11.1)	0.975
LV hypertrophy, No. (%)	24 (12.2)	16 (11.3)	8 (14.8)	0.499
LV diastolic dysfunction, No. (%)	72 (36.7)	45 (31.7)	27 (50)	0.018^*^
H.F with preserved EF, No. (%)	14 (7.1)	6 (4.2)	8 (14.8)	0.024^*^
LV systolic dysfunction, No. (%)	65 (33.2)	48 (33.8)	17 (31.5)	0.758
LV EF, No. (%)	45 (35-50)	45 (35-50)	42.5 (30-50)	0.300
RV systolic dysfunction, No. (%)	25 (12.8)	19 (13.4)	6 (11.1)	0.670
Valvular dysfunction, No. (%)	122 (62.2)	88 (62)	34 (63)	0.898
Tricuspid regurgitation, No. (%)	13 (6.6)	12 (8.5)	1 (1.9)	0.118
Other valvular dysfunctions, No. (%)	109 (55.6)	76 (53.5)	33 (61.1)	0.339
Pulmonary hypertension, No. (%)	27 (13.8)	20 (14.1)	7 (13)	0.839
Pericardial effusion, No. (%)	25 (12.8)	15 (10.6)	10 (18.5)	0.136
**Clinical outcomes**				
Severe cases, No. (%)	126 (64.3)	85 (59.9)	41 (75.9)	0.036^*^
ICU admission, No. (%)	98 (50)	63 (44.4)	35 (64.8)	0.011^*^
ICU stay, median (IQR), day	0.5(0-10)	0 (0-9)	5.5 (0-10.25)	0.032^*^
Hospitalization, median (IQR), day	9.5 (6-15)	9 (6-14)	11 (6-18)	0.144
Invasive mechanical ventilation	50 (25.5)	33 (23.2)	17 (31.5)	0.237
Non-invasive mechanical ventilation	59 (30.1)	40 (28.2)	19 (35.2)	0.339
Mortality	60 (30.8)	42 (29.8)	18 (33.3)	0.631
readmission	13 (6.7)	8 (5.7)	5 (9.3)	0.354

Abbreviation: IQR, interquartile range; LV, left ventricle; HF, heart failure; EF, ejection fraction; RV, right ventricle; ICU, intensive care unit *P* < 0.05

**Table 3:** The descriptive results of ferritin, d-Dimer, and lactate dehydrogenase

**Characteristics**	**Total (n=196)**	**Non-CV complication (n=142)**	**CV complication (n=54)**
Ferritin, median (IQR), mg/L (N = 50)	378 (173-1000)	270 (132-1000)	478.25 (209.25-1633)
D-dimer. Median (IQR), ng/mL (N = 81)	1992 (300-5000)	1190 (105-3510)	2672 (1200-7500)
Lactate dehydrogenase, median (IQR), U/L (N = 98)	606 (423-767)	680 (423-957)	584.5 (405-698.75)

The hydroxychloroquine sulfate was the most common medication used in this investigation (80.1%), yet with no statistical between-group differences (*P* = 0.057). Data showed no remarkable difference between the two groups with regard to either antibiotic or antiviral treatment. Of antiplateletes, aspirin was received by 27% of the cases, and it was significantly higher in patients with CV complication (44.4%) compared to patients with non-CV complication (20.4%) (*P* = 0.001). The consumption rates of anticoagulants, including heparin, and novel oral anticoagulants, were 67.9%, 15.8%, and 0.5% among patients, respectively, with similar rates between the two groups. Of the participants, 19.9% had undergone inotropic treatment, and as expected, the proportion was significantly higher in the CV complication group than the non-CV complication group (29.6% vs 16.2%, respectively; *P* = 0.035), reflecting the high rate of CV complications.

### Cardiovascular examinations

We found ST-segment changes including elevation, depression, and T wave inversion in 19.9% of all patients, with significantly higher frequency in patients with CV complications (33.3% vs 14.8% *P* = 0.004). Other ECG abnormalities concerning the QRS wave and QT interval were comparable (Table 2).

According to echocardiographic data, left ventricular (LV) diastolic dysfunction was the most prevalent abnormality (36.7%), followed by LV systolic dysfunction (33.2%), hypertrophy (12.2%), and dilation (11.2%). There was a 7.1% rate of heart failure with preserved EF and a 12.8% rate of right ventricular dysfunction. The median measure of LV ejection fraction (EF) was 45% (35-50) and the rate of pulmonary hypertension (≥35 mm Hg) was 13.8%. Besides, pericardial effusion was observed in 12.8% of the participants. Valvular dysfunction was categorized into two groups: tricuspid regurgitation (6.6%) and other valvular dysfunctions (55.6%). Patients with CV complications compared with patients without CV complications had a significantly higher proportion of LV diastolic dysfunction and heart failure with preserved EF (50% vs 31.7%, *P* = 0.018; 14.8% vs 4.2%, P = 0.024). Other echocardiographic data were similar between the two groups.

### Complications and clinical outcomes

**Figure 1:**
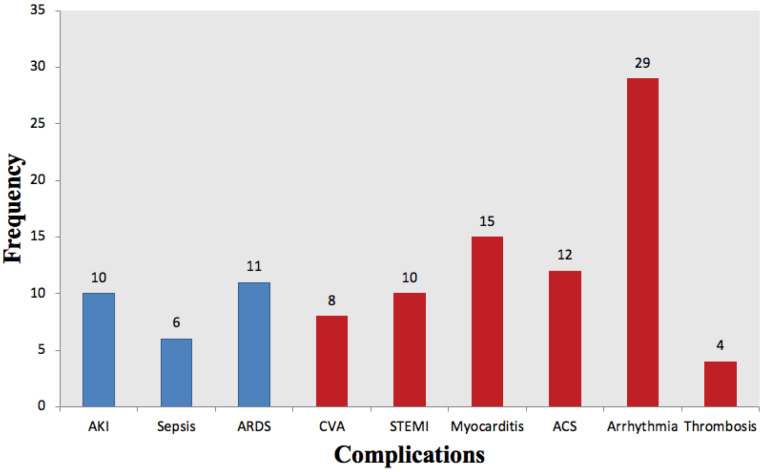
Frequency of cardiovascular and non-cardiovascular complications.

Of the total population, 54 (27.5%) patients had at least one cardiovascular complication. Regarding CV complications, arrhythmia was the most prevalent with a percentage of 14.8%. Given type of arrhythmia, there were 10 (34.4%) atrial fibrillation, 6 (20.6%) bradychardia, 5 (17.3%) premature ventricular contraction, 3 (10.3%) premature atrial contraction, 2 (7%) atrial flutter, 2 (7%) ventricular tachycardia cases and, 1 (3.4%) case of paroxysmal supraventricular tachycardia. The second major CV complication included myocarditis in 15 (7.7%) patients; comprising 6 myocarditis cases with preserved EF. Furthermore, ACS, STEMI, CVA, and DVT had developed in 12 (6.1%), 10 (5.1%), 8 (4.1%), and 4 (2%) patients, respectively. Of the non-CV complication group, ARDS, AKI, and sepsis were observed in 11 (5.6%), 10 (5.1%), and 6 (3.1%) patients, respectively (Figure 1).

AKI, acute kidney injury.; ARDS, acute respiratory distress syndrome; CVA, cerebrovascular accident; STEMI, ST-segment elevation myocardial infarction; ACS, acute coronary syndrome;

Blue columns indicate non-cardiovascular complications and red columns indicate cardiovascular complications.

**Figure 2:**
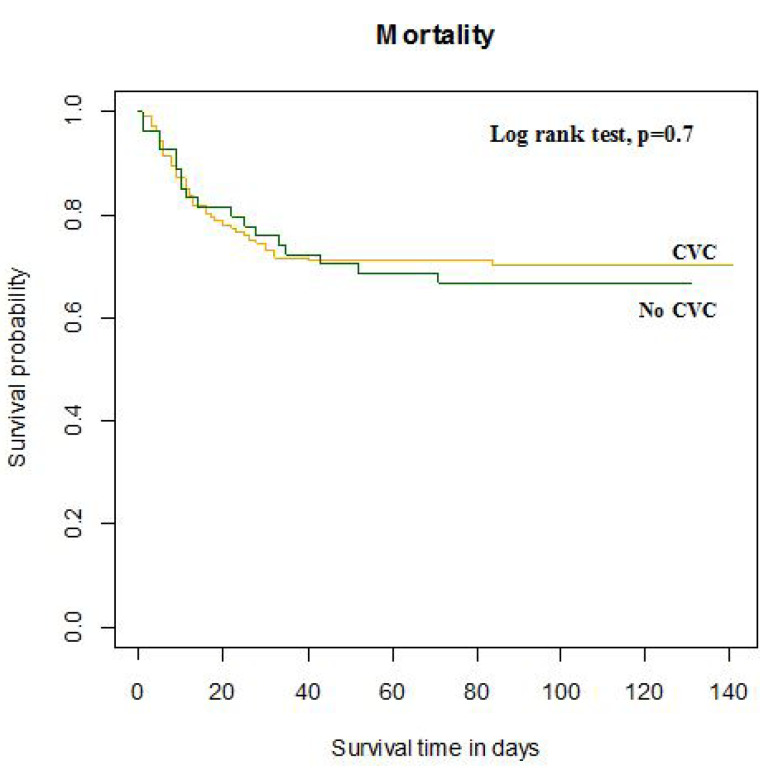
Kaplan-Meier survival curves for mortality from admission to three months after discharge. CVC, cardiovascular complications.

Severe cases comprised nearly two-thirds (64.3%) of our study sample, and there were significantly more severe cases with a CV complication than those without CV complication (75.9% vs 59.9%, *P* = 0.036). Half (50%) of our patients required ICU admission. Also, the median ICU and hospital stay for all patients were 0.5 (0-10) and 9.5 (6-15) days, respectively. A total of 50 (25.5%) patients underwent invasive mechanical ventilation, and 59 (30.1%) patients were treated with non-invasive mechanical ventilation. Among 196 patients, 50 died during their hospitalization and 10 died after discharge, yielding an all-cause mortality rate of 30.8%. Additionally, 13 (6.7%) patients were readmitted throughout the three-month follow-up period. The ICU admission percentage (64.8% vs 44.4%, *P* = 0.011) and the median ICU stay (5.5 days vs 0 days, *P* = 0.032) were notably higher in patients with CV complications. Other clinical outcomes including mortality were comparable between the two groups. A similar result was observed in Kaplan-Meier survival curves for mortality (Figure 2).

### Risk factors for mortality

The multivariate Cox regression analysis revealed that increasing age, history of heart failure or cancer/autoimmune disease, disease severity, in-hospital treatment with interferon beta-1a, and arrhythmia were the independent predictors of all-cause mortality during and after hospitalization (Table 4).

**Table 4:** Multivariate Cox regression analysis on the risk factors associated with mortality in patients with COVID-19

**Variables**	**HR (95% CI)**	***P*** ** value**
Age, y	1.02 (1-1.04)	0.038
Heart failure	2.03 (1.14-3.60)	0.016
Cancer/autoimmune disease	5.57 (2.08-14.92)	0.001
Arrhythmia	2.02 (1.10-3.72)	0.024
Severity	5.36 (2.37-12.15)	0.000
Interferon beta 1-a	1.76 (1.02-3.03)	0.043

We used a univariate Cox regression analysis to assess possible factors associated with higher mortality. Age, history of heart failure, history of cancer/autoimmune disease, acute respiratory distress syndrome, arrhythmia, in-hospital treatment with inotropic, atazanavir, heparin, and interferon beta 1-a, antibiotic therapy, and severity were significant factors with a P-value < 0.1 that further entered in the multivariate analysis.

## Discussion

To our knowledge, the present retrospective cohort study is one of the very few studies that has comprehensively addressed CV complications and related outcomes in COVID-19 patients. Previous works have almost exclusively focused on cardiac injuries without a definitive diagnosis. We reviewed data of 196 patients admitted with laboratory-confirmed COVID-19 requiring a cardiologist consultation. CV complications as a common outcome occurred in 27.6% of all patients. In patients with CV complications, arrhythmia (14.8%) was the most common complication associated with an increased risk of death during and after hospitalization.

Acute cardiac injury as conventionally characterized by high-sensitivity troponin I (hs-TnI) > 99th percentile upper reference limit is one of the most prevalent pathologies (ranging from 7.2% to 19.7%) found in the context of COVID-19 and occurs in the setting of myocardial tissue damage.^3,6^ The literature has highlighted cardiac injury as a strong predictor of adverse clinical outcomes in COVID-19, including the severity of the viral disease, ICU admission, and mortality. However, the literature fails to specify the type and prevalence of injuries.^3,7,8^ The study of Shi et al showed that 19.7% of 416 patients experienced a cardiac injury, which held a direct correlation with higher severity and an independent association with mortality (adjusted HR 4.26).^7^ Another study with 187 patients reported a heightened in-hospital mortality rate in cases with high Troponin-T levels (37.5%) compared to those with a normal level (7.62%). This difference was even more profound when a high Troponin-T level was accompanied by an underlying CV condition (69.44%).^6^ Wang et al found a higher proportion of myocardial injury in severe patients (22.2% vs 7.2%) and confirmed its relationship with all-cause mortality.^3^ We also observed a notable increase in the rate of elevated Troponin-I and NT-proBNP in patients with CV complications, which, per se, indicates a higher prevalence of acute cardiac injury in this group. In line with previous reports, severity, ICU admission and stay were significantly higher in patients with CV complications than those without. However, contrary to most reports, there were no significant between-group differences for mortality.

COVID-19 is a systemic disease mostly manifested with pulmonary symptoms. Yet, several mechanistic pathways contribute to a wide range of CV involvements. Inflammation is the primary recognized mechanism. After activation of pulmonary macrophages, a large number of cytokines are released, which enter into the circulatory flow. As such, both inner and adoptive immune systems get activated, leading to the infiltration of mononuclear cells into the myocardium.^9^ In this regard, multiple reports have shown acute myocarditis as a serious complication of COVID-19; nevertheless, the prevalence has remained uncertain.^10-12^ Consistently, we found 15 cases of myocarditis and higher levels of ESR and CRP in patients with CV complications.

Alternatively, fever and sympathetic nervous system overactivation increase cardiac muscle demand for oxygen.^13^ On the other part, oxygen supply to the myocardium is decreased following pulmonary damage, which may be aggravated by cytokines induced-hypotension.^13,14^ A recent report has shown that alveolar macrophages may migrate to the heart while they carry SARS-CoV-2, resulting in a local inflammation that is implicated to destabilize the pre-existing atheromatous plaques in the lumen of coronaries.^15^ COVID-19 induces a hypercoagulable state caused by overexpression of adhesion molecules and tissue factors, increased level of prothrombotic factors, and thrombocytes. Moreover, the disruption of myocardial circulation is boosted by the dysfunction of the SARS-CoV-2 receptor (ACE2) on pericytes, which play an important role in maintaining endothelial function.^16^ thereby leading to common ischemic and/or thrombotic events seen in COVID-19.^13^ Similarly, ischemic/thrombotic events (17.3%) are the most frequent complication occurring in our study when we consider MI, ACS, CVA, and DVT as a single subgroup.

In the present study, the LV diastolic dysfunction (36.7%) was the most common condition followed by the LV systolic dysfunction (33.2%) and, less commonly, the right ventricular (RV) systolic dysfunction (12.8%). The study of Szekely et al examined 100 consecutive patients with COVID-19 with complete echocardiography within 24 hours of admission, finding that the most common pathology was RV dysfunction (39%), while LV diastolic and systolic dysfunctions were present in 16% and 10% of cases, respectively. LV diastolic and RV dysfunctions were impaired in the majority of patients, and only RV dysfunction was linked to higher Troponin levels.^17^ Another study investigated 1,216 patients from 69 countries, reporting that 55% of cases had an abnormal echocardiogram and that the rate of LV dysfunctions (39%) was greater than that of RV dysfunctions (33%). However similar to the previous study, only RV dysfunctions were independently associated with increased levels of cardiac markers and higher disease severity.^18^ In contrast, we observed that LV diastolic dysfunction was significantly more common in patients with CV complications while the difference regarding RV dysfunction was not remarkable. The contradiction between earlier reports and the present study can be explained by our higher prevalence of severe cases and the different pool of patients (patients with suspicioned CV complications). We presume that new-onset LV dysfunctions during the clinical course may be a more specific predictor of CV complications, while RV dysfunctions is caused by pulmonary hypertension due to more severe lung infections and may be linked to increased release of cardiac markers as a result of increased right atrial wall pressure. However, this hypothesis needs further investigation.

Drawing upon the findings of Dweck et al.’s study, where performing echocardiography in COVID-19 led to changes in management and level of care in 33% of the cases, including ICU admission and hemodynamic support, we may justify the comparable mortality rate between the two groups of our study by the higher level of care, including ICU admission and stay, received by patients with CV complications.^18^

Our results cast new light on arrhythmia as an independent risk factor for death. Although studies that specifically aim to investigate arrhythmia in COVID-19 are lacking, there is limited evidence showing the association of arrhythmia with cardiac injury and disease severity.^3,6^ In the study of Wang et al arrhythmia was the second serious complication after ARDS, and its rate was increased in patients admitted in ICU (44.4%) compared to those in a general ward (16.7%).^3^ Also, a cohort study with 393 COVID-19 patients reports a higher rate of atrial arrhythmias among individuals with invasive mechanical ventilation (17.7%) versus those with non-invasive ventilation (1.9%).^19^ Similarly, an analysis on 115 cases (69 in the ICU and 46 in the general ward) observed new-onset atrial tachyarrhythmia in 16.5% of the study population, all admitted in the ICU.^20^ Systemic and myocardial inflammation, metabolic disturbances, ischemic events, and overactivation of the sympathetic system can be potential underlying mechanisms that contribute to the incidence of arrhythmia. Even after discharge, cardiac injury may cause scar formation in the myocardium and subsequent conduction disorders, leading to the incidence of out-of-hospital arrhythmia and sudden cardiac death as proved by the study of Baldi et al in Italy who showed an increase (52%) in out-of-hospital cardiac arrests compared to the same timeframe before the pandemic.^21^

There are conflicting results upon administration of Interferon in COVID-19 patients. As a biological antiviral agent, Interferon beta 1-a is now a leading candidate for the treatment of COVID-19 patients due to its beneficial effects seen in multiple reports and clinical trials.^22,23^ On the other hand, some reports have failed to show any association between Interferon use and improved clinical outcomes. Alternatively, some reports mention increased mortality and delayed recovery upon late administration of Interferon-alpha, while its early administration was accompanied by improved outcomes.^24,25^ In our study, in-hospital treatment with Interferon beta-1a was a strong predictor of all-cause mortality. The clinical course in COVID-19 consists of two phases: 1-early viral replication phase and 2- late cytokine release phase. From this standpoint and according to the results of previous reports, it seems that Interferon-based treatment is effective when administered in an early phase and for less severe patients. Unfortunately, the timing of administration was not evaluated in our study. However, there were more severe cases in our study population.

Our study had some limitations. First, the study design was retrospective. Second, we only included patients whose attending physician had requested for cardiology consultation. Although both the attending physician and the cardiologist remained unchanged during the study period, the decision for whether to request a consultation or not is a potential bias. Future investigations with larger sample sizes and on consecutive patients are recommended to further confirm the results of our study.

## Conclusion

In summary, it would appear that CV complications commonly occur with a wide range in COVID-19 patients. Arrhythmia was the most common complication observed in the present study. Patients who experience clinical deterioration and/or changes in ECG findings or cardiac markers should be evaluated for further underlying CV complications. Diagnosis of such patients has clinical importance for early management, treatment, and referral for intensive care to improve prognosis. Our analyses confirmed older age, history of heart failure or cancer/autoimmune disease, in-hospital treatment with interferon beta-1a, greater severity, and incidence of arrhythmia as independent risk factors for all-cause mortality during and after hospitalization.

## Competing interests

None to declare.

## Ethical Approval

The local university’s ethics committee approved the study protocol with approval number of IR.MUBABOL.REC.1399.182.

## Funding

This research did not receive any specific grant from funding agencies in the public, commercial, or not-for-profit sectors.
